# Acquired Renal Amyloidosis in a Patient With X-Linked Hyper-IgM Immunodeficiency With Novel Hemizygotic Pathogenic Variant in CD40LG Gene

**DOI:** 10.1155/crin/6664645

**Published:** 2025-09-02

**Authors:** Daniel Celis-Giraldo, Deider Steeven García-Villamizar, Camilo Parra-Amaris, Diana Carolina Gutiérrez-González, Daniel Rodríguez-Peralta

**Affiliations:** ^1^Internal Medicine Program, Universidad Militar Nueva Granada, Bogotá, Colombia; ^2^Department of Internal Medicine, Hospital Militar Central, Bogotá, Colombia; ^3^Department of Nephrology, Hospital Militar Central, Bogotá, Colombia; ^4^Department of Pathology, Hospital Militar Central, Bogotá, Colombia

**Keywords:** CD40LG gene, hyper-IgM immunodeficiency, nephrotic syndrome, renal amyloidosis

## Abstract

**Introduction:** Renal AA amyloidosis with X-linked hyper-IgM immunodeficiency is rare diseases, and their simultaneous presentation in the same patient is exceptional.

**Case Presentation:** We present a case of renal AA amyloidosis in a 20-year-old man with nephrotic syndrome and reduced glomerular filtration rate (GFR). Clinically, serologically, histopathological, and genetically, we confirm renal amyloidosis in the presence of X-linked hyper-IgM syndrome; in turn, we detected a new hemizygous pathogenic variant in the CD40L gene (c.345delA).

**Conclusion:** Our hypothesis suggests that these conditions predisposed the patient to a combined (cellular and humoral) immunodeficiency, leading to recurrent infectious episodes throughout his life, ultimately resulting in renal amyloidosis due to deposition of serum amyloid protein.

## 1. Introduction

Amyloidosis is a disorder characterized by abnormal protein folding, which leads to extracellular accumulation of insoluble fibrillar proteins known as amyloid fibrils [1]. Misfolding proteins form oligomers, aggregates, and amyloid fibrils with β-sheet structures that can disrupt tissue architecture and function. This process results in a wide range of clinical manifestations, from localized organ dysfunction to systemic multiorgan failure [2, 3].

The most common types of amyloidosis include AL amyloidosis, characterized by the production of light chains associated with monoclonal plasma cell populations; transthyretin (TTR) amyloidosis, where normal TTR aggregates predominantly causing cardiomyopathy; and AA amyloidosis, which arises from the deposition of acute phase reactant serum amyloid A (SAA) protein, that is, a highly conserved precursor synthesized by hepatocytes in systemic and inflammatory diseases [4, 5].

AA amyloidosis has an incidence rate about 1 to 2 cases per million person-years, with a mean age of onset of 54 years [5]. Out of 16,175 tissue samples analyzed for amyloidosis typing using mass spectrometry over an 11-year period in Mayo Clinic, AA type was identified in only 2.9% of cases [6]. The association between AA amyloidosis and primary immunodeficiencies (PIDs) is rarer and has been documented through case series and autopsy studies [7–9]. In the largest European cohort, in 42 cases reported from 1949 to 2019 of AA amyloidosis, only 3 was report in patients with hyper-IgM immunodeficiency (HIM) [10].

These patients present with multiple infections, mainly bacterial pneumonias that begin during adolescence or early adulthood, the lack of clinical suspicion, without adequately studying the causes of these repeated infections, leads to late diagnosis and treatment of immunodeficiency. The clinical course is characterized by chronic lung infections, as evidenced by bronchiectasis in more than half of the patients, eventually leading to the development of nephrotic syndrome revealing AA amyloidosis until 16 years later. The delay from immunodeficiency symptoms to amyloidosis diagnosis is ranged from 11.17 to 23.17 years [10]. In this group of patients, renal involvement represents around 80% of the disease, being the most frequent manifestation. In some cohorts, approximately half of the patients will present end-stage renal disease (ESRD) at diagnosis, with a variable time to progression of around 26 months [7]. Among those with renal AA amyloidosis, 95% exhibit proteinuria, and 50% were present with nephrotic syndrome, with a 24 h mean proteinuria of 3.9 g [5].

The precise mechanisms by which these precursors localize and assemble into amyloid fibrils are not fully understood. However, it is thought that slightly misfolded SAA proteins evade the cell's quality control systems, resulting in the exposure of normally hidden hydrophobic regions. These exposed regions make the proteins more likely to interact with similar molecules, leading to aggregation. A major factor in the toxicity of amyloid fibrils is their ability to disrupt cellular lipid membranes [11].

Several studies have elucidated that the binding of 1-anilinonaphthalene-8-sulfonic acid (ANS) to oligomers formed by misfolded proteins reveals the presence of hydrophobic sites on their surface. In turn, these exposed regions are a particular feature of toxic oligomers and are capable of altering the integrity of membranes [11, 12]. In essence, the lipid bilayer structure of the membrane is destabilized by the presence of oligomers, and therefore, the altered cell membrane allows the transmembrane ionic charge imbalance to promote the influx of calcium ions (Ca2+). This electrochemical phenomenon in turn triggers a cascade of deleterious effects, such as the generation of reactive oxygen species (ROS) and the activation of apoptosis or other forms of cell death. At the renal level, this pathological mechanism causes loss of nephron mass, leading to a progressive deterioration of renal function characteristic of amyloidosis [12].

Besides, HIM is an uncommon PID characterized primarily by an inability to produce high-quality immunoglobulin of any isotype apart from IgM (usually with relatively normal to elevated levels of IgM). When a new antigen is presented to the immune system, the first antibodies made are generally IgM [13]. B cells then continue to differentiate into IgG (the most abundant), IgA (important in mucosal immunity), or IgE (involved with allergy and parasite protection) producing cells, which provide robust protection in tissues and mucosal surfaces [14]. Most of the HIM presentations described have been a defect in the TNFSF5 gene on the long arm and band 26 of the X chromosome [15].

CD40 ligand (X-linked) is a T-cell surface protein that binds CD40, its receptor, on antigen-presenting cells (APCs) and nonimmune cells. The sort that splits after switching on the mechanism because a genetic defect provides aberrant CD40L protein with a broad range of harmful impacts barrels immune response regulation. It predominantly impacts the interaction between CD40L and CD40 on B cells, which is an essential costimulatory signal necessary for the differentiation of B cells into plasma cells to generate antibodies via class-switch recombination (CSR) [16]. CD40L proteins with defects that inhibit CSR led to normal or high serum IgM but low or undetectable IgA, IgE, and IgG. This pathological process, in turn, leads to a lack of antibody diversity such that it profoundly weakens the humoral response and predisposes the patient to recurrent infections [17].

It is known that altered CD40L signaling through monocytes and dendritic cells impairs T cells and thus further dampens cellular immune responses resulting in risk to opportunistic infections [18]. However, most patients (over 90%) are symptomatic by age 4 years, usually in infancy with recurrent bacterial upper and lower respiratory tract infections, opportunistic infections, and prolonged recurrent diarrhea, infectious or noninfectious etiology [19].

## 2. Case Presentation

A 20-year-old male patient with a history of asthma, allergic rhinitis, and common and variable immunodeficiency diagnosed at age of four was receiving monthly immunoglobulin G supplementation, with poor adherence to therapy over the past 6 months. Family history includes a younger brother with X-linked recessive HIM due to a likely pathogenic variant in the CD40LG gene c.345delA; p.Gly116Val fr ∗ 12 in hemizygosity and a variant of uncertain clinical significance in the C3 gene c.746a > g; p.Lys249Arg in heterozygosity with uncertain clinical significance, who sadly passed away a year ago as a result of complications related to a bone marrow transplant.

The patient presents to the emergency department with a 3-day history of respiratory symptoms, headache, and fever, along with recent onset of foamy urine in the last month. The patient had history of repeated respiratory, gastrointestinal, and aural infections during the first 5 years of life, and again in the last year with recurrent infections requiring emergency room visits, last hospitalizations were six months and a month ago for 2 episodes of community-acquired pneumonia; totaling, there are approximately 20 infection episodes with emergency room visits and many episodes with outpatient treatment throughout life.

Upon admission, the patient presented with a hemogram showing neutropenia, lymphocytosis, mild low-volume anemia, thrombocytosis, elevated C-reactive protein, and high creatinine at 1.65 mg/dL resulting in an estimated glomerular filtration rate (eGFR by CKD-EPI equation) of 60 mL/min/1.73 m^2^, high blood urea nitrogen (BUN) levels, and proteinuria in urine analysis without hematuria or leukocyturia. IgG and IgA levels were below the detection limit, with IgM at normal levels (101.98 mg/dL) (Table 1). Further testing included negative results for respiratory microorganisms in BIOFIRE FILMARRAY Pneumonia Panel plus, negative sputum acid-fast bacilli smears, and negative XpertMTB/RIF-Ultra for *Mycobacterium tuberculosis*. Chest tomography revealed bilateral paratracheal and perivascular lymph node enlargement, smooth thickening of bronchial walls at bilateral and distal perihilar levels on the right lung associated with cylindrical bronchiectasis, and centrilobular nodules in the medial and lateral segments with an adjacent area of alveolar space occupation towards the parahilar region. The left lung showed centrilobular nodules associated with cylindrical bronchiectasis at the posterior superior and basal segments. A complete follow-up blood count and normal lung biopsy were performed. The patient received antimicrobial treatment resulting in symptom resolution.

**Figure 1:**
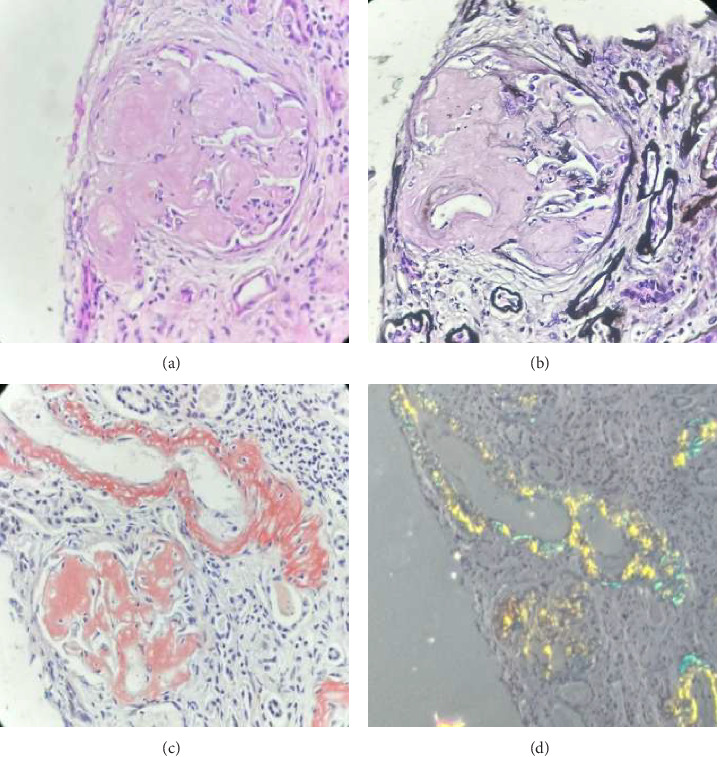
Amyloid A amyloidosis (AA). (a) Photomicrograph of a glomerulus at 400x magnification with Hematoxylin & Eosin staining, showing mesangial expansion and the presence of pale eosinophilic, amorphous material, also affecting the glomerular basement membranes. (b) Photomicrograph of a glomerulus at 400x magnification with methenamine silver staining, showing mesangial expansion and the presence of pale amorphous material, which appears negative, also affecting the walls of arterioles in the vascular pole. (c) Photomicrograph of a glomerulus at 100x magnification with Congo Red staining, showing mesangial expansion and reddish amorphous material, also affecting the walls of adjacent arteries. (d) Photomicrograph of the same glomerulus at 100x magnification with Congo Red staining under polarized light, showing apple-green birefringence with dichroism in areas with deposits.

**Figure 2:**
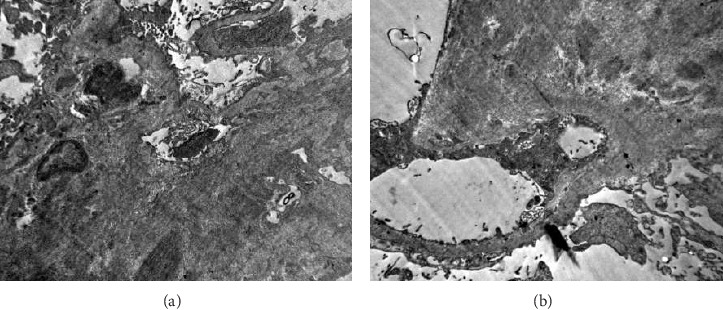
Amyloid A amyloidosis (AA). (a) Electron micrograph of a glomerulus showing a large number of nonbranching fibrils in the mesangium, measuring between 10 and 12 nm in width, consistent with amyloid. (b) Electron micrograph of a glomerulus showing a large number of nonbranching fibrils in the mesangium, measuring between 10 and 12 nm in width, consistent with amyloid; this material extends into the basement membranes of the capillary loops.

During hospitalization, the patient presented bilateral soft edema in the lower limbs. Proteinuria was detected in the urine dipstick test, leading to quantification and measurement of proteinuria revealing 6.34 g in 24 h. Further studies showed hypoalbuminemia and hypercholesterolemia, leading to a diagnosis of nephrotic syndrome with a KDIGO II acute renal injury over established chronic kidney disease (CKD). A renal biopsy was performed, showing glomerular changes such as altered architecture and diffuse mesangial expansion with findings suggestive of amyloid deposits, confirmed by their red congo birefringence under polarized light. Immunofluorescence studies ruled out the presence of immunoglobulin or complement deposits (shown in Figure 1). Electron microscopy revealed amyloid fibrils consistent with a diagnosis of renal amyloidosis (shown in Figure 2). Additional diagnostic tests included elevated SAA (48.5 mg/dL [negative < 6.4]), normal serum beta-2 microglobulin, normal immunofixation in blood and urine, and normal measurements of lambda and kappa free chains in blood and urine. Urine protein electrophoresis showed a decrease in the albumin zone with a polyclonal increase in the alpha 2 zone, indicative of elevated inflammatory markers (Table 1). Peripheral flow cytometry shows normal levels of CD3+, CD4+, and CD8+ T lymphocytes. Bone marrow cytology and flow cytometry phenotype were normal. Then, the patient developed diarrhea during hospitalization, leading to a colonoscopy with a colon and ileum biopsy, which ruled out inflammatory bowel disease. Subsequently, the patient developed bacteremia due to *Moraxella osloensis*, which was successfully treated with appropriate antibiotics.

A diagnosis of renal AA amyloidosis was considered. Given the early age of onset and family history, the patient was evaluated by medical genetics, who decided to perform whole exome sequencing using next-generation sequencing (NGS) to rule out additional variants that could explain the patient's condition.

The exome sequencing was conducted on a DNB-SEQ400 massive sequencer using an MGI-V5 exome library. The genes were analyzed with an average coverage of over 98% and a minimum depth of 20x. A new pathogenic frameshift hemizygous variant was identified in the CD40LG gene that may support the clinical suspicion of X-linked HIM: CD40LG (NM_000074.3): c.345del; p.Gly116ValfsTer12. The patient showed improvement with supplementation of subcutaneous immunoglobulin G at a dose of 45 g, optimized every 15 days, completed the vaccination schedule, and received a multimodal antiproteinuric treatment with empagliflozin 10 mg orally o.d., enalapril 5 mg orally o.d., and spironolactone 25 mg orally o.d., leading to improvement in proteinuria and stability of the GFR without the need for further hospitalizations due to disease decompensation, but without normalization of creatinine levels at baseline, meeting CKD criteria.

## 3. Discussion

Here, we present a case of renal AA amyloidosis in a young patient related to X-linked HIM syndrome. There are fewer than five reported cases of this worldwide [10]. The early age of presentation in our case is notable, considering the average age of presentation is 33 years; additionally, the proteinuria was quantified at 6.3 g of proteins, nearly double the usual presentation average, which may correspond to the degree of renal involvement in our patient. However, our patient is not in the terminal stage as described in up to 53% of cases of renal amyloidosis in some cohorts [7], but with risk of progression to kidney failure requiring dialysis or transplant of 55.67% at two years and 95.71% at five years according to kidney failure risk score (KFRE).

Our hypothesis is based on the phenomenon of recurrent infections experienced by the patient since childhood, triggering a chronic inflammatory response mediated by TNF-α, IL-1, and IL-6, which may predispose the liver, macrophages, and smooth muscle to maintain an inflammatory response that conditions the production of serum amyloid P (SAP) component and glycosaminoglycans (GAGs), precursors of amyloids that ultimately deposit in multiple organs [20]. Our patient had multiple respiratory and gastrointestinal infections, about 20 clinically relevant episodes, facilitated by nonadherence to therapy with intravenous immunoglobulin G (IVIG), and the presence of bronchiectasis, known as an essential risk factor in recurrent infections; also, the phenomenon described from immunodeficiency presentation to amyloidosis diagnosis, can take up to 16 years in larger series, as evidenced in our case study [10].

This case is remarkable for its early presentation with significant renal involvement, in the context of a family history in which the patient's younger brother had HIM associated with a pathogenic CD40L gene variant and a C3 gene variant of uncertain clinical significance, leading to a fatal outcome following complications of salvage bone marrow transplantation. Whole exome sequencing was performed by NGS identifying a novel mutation not previously reported in the CD40L gene. CD40L of activated CD4+ T cells interacts with its receptor on both APCs and nonimmune cells [21]. Therefore, an aberrant CD40L protein can have broad damaging effects. Specifically, when CD40L and CD40 interact on B cells, it delivers an essential signal that supports the maturation of B cells and the production of antibodies by facilitating CSR [22]. Malfunctioning CD40L proteins hinder CSR, resulting in normal or increased levels of serum IgM, alongside reduced or undetectable levels of IgA, IgE, and IgG, as seen in our patient. This lack of antibody diversity weakens the humoral response, rendering patients susceptible to recurrent bacterial infections. Impaired CD40L signaling also affects APCs such as monocytes and dendritic cells, further compromising T-cell activation and increasing susceptibility to opportunistic infections [18].

**Figure 3:**
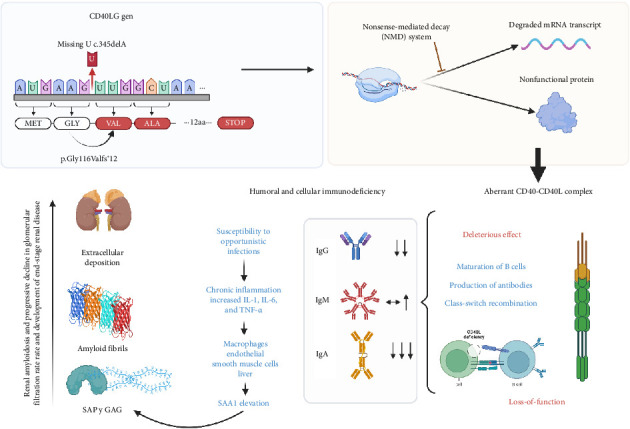
The diagram shows a detailed molecular pathophysiological cascade triggered by a new CD40LG gene mutation that results in a nonfunctional CD40-CD40L complex, driving immunodeficiency, chronic inflammation, and systemic complications like renal amyloidosis (created at biorender.com).

In the present case, a hemizygous pathogenic variant was identified in the CD40LG gene (NM_000074.3), involving a deletion of a nucleotide at position 345 in the cDNA, exon 3 of the gene (c.345delA), resulting in a *frameshift* change leading to a premature stop codon at amino acid 127 in a 261-amino acid protein (p.Gly116ValfsTer12). This variant likely produces a degraded mRNA transcript by the nonsense-mediated decay (NMD) system or a nonfunctional protein, indicating an expected deleterious effect. Loss-of-function variants are a known mechanism of pathogenicity for this gene. This variant is not reported in ClinVar (https://www.ncbi.nlm.nih.gov/clinvar/), The Human Gene Mutation Database (HGMD) (https://www.hgmd.cf.ac.uk/), or Leiden Open Variation Database (LOVD) (https://www.lovd.nl/). This finding represents the first case of its kind globally where a *frameshift* variant predisposes to X-linked immunological disorders that clinically determine susceptibility to chronic infections that promote the development of long-term complications such as amyloidosis (shown in Figure 3).

So far, nearly 85 pathogenic variants of CD40L have been reported in the NCBI ClinVar database (as of 25 May 2025). From these, 72 are pathogenic variants in patients with X-linked hyper-IgM syndrome and are associated with recurrent infections, malignancies, carcinoid tumors, and sclerosing cholangitis (23, 24). However, no cases related to renal AA amyloidosis have been reported.  Table 2 presents several variants identified in comparison with the novel variant described in this report, along with their respective associated clinical phenotypes.

In terms of treatment, several targeted therapies like eprodisate [23] or anticytokines [27, 32, 33] have been attempted without definitive outcomes. Notably, anakinra seems to show effectiveness in treating nephrotic syndrome in individuals with AA amyloidosis and autoinflammatory disease linked to CIAS1/NALP3/cryopyrin gene mutations [34]. Regrettably, it lacks approval from regulatory authorities in Colombia. In this sense, specific therapy to renal AA amyloidosis is to address the primary cause by treating any infection or chronic inflammation, and long-term suppression of amyloid is crucial to improve renal outcomes. A complete response has been defined as normalization of SAA (< 10 mg/L) and resolution of symptoms and exacerbations, whereas a partial response was defined as improvement without normalization of SAA and/or an improvement in disease symptoms [35]. Thus, requiring long-term amyloid measurements is to determine inflammatory control and gradual regression of deposits for improved renal function.

Based on our experience and evidence-based management of AA amyloidosis, nephroprotective strategies, including the use of renin-angiotensin system inhibitors such as ACE inhibitors or ARBs and sodium-glucose cotransporter 2 inhibitors (SGLT2i), are recommended to reduce proteinuria and slow renal decline. Adult vaccination is essential to minimize infection-related inflammatory flares; pneumococcal, meningococcal, *Haemophilus influenzae* type b, annual influenza vaccines, and COVID-19 are typically indicated. In patients with X-linked hyper-IgM syndrome, IVIG replacement therapy at higher-than-standard doses may be warranted to ensure adequate serum IgG levels and reduce infection frequency. Rigorous infection prevention measures—including prophylactic antibiotics, early treatment of infections, and regular monitoring—are critical in reducing the inflammatory burden that drives SAA overproduction. Lastly, although evidence is still emerging, anti-inflammatory dietary interventions—emphasizing omega-3 fatty acids, reduced intake of processed foods, and high consumption of fruits, vegetables, and whole grains—may provide supportive benefit by modulating systemic inflammation and potentially influencing amyloidogenic pathways.

However, prognosis is poor with an estimated survival of 6–9 years, with progressive decrease in GFR and increased creatinine and proteinuria, leading to the development of ESRD and sometimes cardiac and hepatic involvement [5].

While this case report provides meaningful insights into the rare co-occurrence of renal AA amyloidosis and X-linked hyper-IgM syndrome, it is important to acknowledge the inherent limitations of single-patient studies. The clinical observations and interpretations presented here may not be generalizable to the broader population of individuals with similar immunodeficiencies. Additionally, the absence of extended follow-up data limits our ability to assess the long-term effects of therapeutic interventions on disease progression and renal function. Future research—including larger case series and mechanistic studies—is essential to deepen our understanding of the relationship between PIDs and systemic amyloid deposition.

In conclusion, renal amyloidosis should be suspected and presieved in patients presenting immunodeficient syndromes, and what is more in patients with proteinuria or worsening renal function in the second decade of life to avoid delays in treatment. Treatment is based on long-term suppression of amyloid levels to improve renal outcomes, along with vaccination, immunoglobulin G, and antiproteinuric therapy. Our group reports a new pathogenic hemizygous variant in the CD40LG gene, which has a deleterious effect on the transcription of essential proteins for an effective immune response against infections, leading to cellular and humoral immunodeficiency predisposing to multiple infections with subsequent generation of serum amyloid and its deposition in multiple organs, mainly the kidney.

## Figures and Tables

**Table 1:** Patient follow-up variables during hospitalization and subsequent outpatient follow-up.

Test (unit)	Normal values	09-Feb	11-Feb	16 Feb	19-Feb	20-Feb	22-Feb	25-Feb	04-Mar	20-Mar	31-May	26-Jun	28-Jul
Hemoglobin (g/dL)	13.2–16.6	12.2	13.9	11	11.5	10.2		11.5		9.9		10	9.5
Platelets (10^3^/mm^3^)	150–450	561	463	801	871	730		125		373		554	670
Neutrophils (10^3/μL)	2.25–8.48	1	0.7	1.6	14.3	9.73		4.88		0.53		2.41	0.94
Lymphocytes (10^3/μL)	0.9–4.2	3.57	3.38	6.43	2.52	1.6		0.9		1.64		1.39	2.51
Creatinine (md/dL)	0.01–1.1	2.03	1.85	1.6	1.82	1.65	1.16	1.41	1.25	1.48		2.82	5.4
BUN (mg/dL)	0.25–0.3	33.5	31.5	15.2	30	32.7	29	31	29	24		30.6	40.7
GFRe (ml/min/1.73 m^2^)	≥ 90	47	53	63	54	61	92	73	85	69		32	15
Albumin (mg/dL)	0.25–0.8		1.5		1.9		1.9	1.7	1.8	1.6		1.9	1.9
Proteinuria 24 h (mg/24 h)	5–248		6342										
Total cholesterol (mg/dL)	80–200		251										
Triglycerides (mg/dL)	< 150		202							286			
CRP (mg/dL)	0–0.5	20.6						5.3		1.2			
IgG (mg/dL)	0.1–1.8		< 16.2						109	148	314	113	
IgM (mg/dL)	35–242		101						70.9	79	59	85	
IgA (mg/dL)	84–499		< 2.05						< 2.05	< 2.05	< 2.05	< 2.05	
IgE (mg/dL)				0									
b2-microglobulin(mg/dL)	0.8–2.3						4.5						
Ferritin (ng/mL)	30–400					105				191			299
Fibrinogen (mg/dL)										900			

Abbreviations: CRP, C reactive protein; IgA, A immuoglobulin isotype; IgE, E immuoglobulin isotype; IgG, G immuoglobulin isotype; IgM, M immuoglobulin isotype.

**Table 2:** Pathogenic variants in the CD40LG gene and their associated clinical phenotypes.

Genetic variant	Molecular consequence/variant type	Age at onset	Clinical phenotype	Country	IgG/IgA/IgM	Clinical course/manifestations	Source (reference)
c.714delT (p.Phe238Leufs ∗ 4)	Frameshift	Infancy (mean of 3.8 years)	Classic X-HIGM	Iran	↓IgG/↓IgA/↑IgM	Recurrent pneumonia	[23]

c.121_122insCAGCAC	Nonsense, insertion	28 years	X-linked Hyper-IgM	Brazil	↓IgG/↑IgM/↓IgA	Cryptococcal meningoencephalitis, recurrent respiratory infections	[24]

c.429_429 delA (p. G144DfsX5) c.500 G > A (p.G167E) c.156 G > C (p.K52 N)	Missense, deletion	Median age 3.5 years	X-linked hyper IgM syndrome	India	↓IgG, ↓IgA, ↑IgM	Sinopulmonary infections (80%) and diarrhea (50%). Sclerosing cholangitis and necrotizing fasciitis were noted in one patient each. Neutropenia (50%).	[25]

c.257delA (p.E86Gfs ∗ 9)	Frameshift	Infancy	Classic X-HIGM	China	↓IgG/↓IgA/↑IgM/IgE normal	Severe pneumonia secondary to HCMV infection and hypogammaglobulinemia.	[26]

c.107T > G (p.Met36Arg)	Missense, single nucleotide variant	Variable	X-linked hyper-IgM syndrome (XHIM)	U.S.	↓IgG/↓IgA/↑IgM	PCP was reported in 42% and neutropenia in 62%. Persistent cholangiolitis and liver cirrhosis. Five patients developed tumors of the gastrointestinal tract, including hepatic/pancreatic carcinoid, bile duct carcinoma, and adenocarcinoma of unknown origin. Parvovirus B19–induced pure red cell aplasia.	[27]

p.S89TfsX6	Frameshift nonsense	7 years	X-linked hyper-IgM syndrome (XHIM)	Iran	↓IgG/↓IgA/↑IgM	Pneumonia 76%, otitis media 55%, lymphoproliferation 42%, neutropenia 57%, and chronic diarrhea 52%	[28]

Del 347 A (Fs 108, stop at 127) Del ACATGT (Del 37Asp, 38Ser) Del 366^a^ (Fs122, stop 127)	Nonsense/missense	Variable	Classic to mild adult-onset phenotypes	Taiwan	↓IgG/↓IgA/↑IgM or variable	Ileal perforation Parainfluenza type III pneumonia Not related	[29]

(c.172delA, c.A229T, c.C478T) (c.A506G) [c.346 + 2(T⟶C), c.289-1(G⟶C), c.346 + 1(G⟶T)]	Nonsense/missense/splice sites	2.6	X-linked hyper-IgM syndrome (XHIM)	India	—	Lower respiratory tract infections (85), recurrent diarrhea (57%), otitis media (28%), lymphadenopathy (28%), arthritis (14%), skin lesions (14%), and pulmonary tuberculosis (28%).	[30]

c.156+2T > A c.436_438delTAC c.654C > A	Splicing/nonframeshift deletion/stop-gain	Variable	X-linked hyper-IgM syndrome (XHIGM)	Vietnam	↓IgG/↓IgA/↑IgM or normal	Otitis media, recurrent sinopulmonary, oral ulcer, neutropenia, and recurrent diarrhea Lung abscess, Crohn's disease, and antiphospholipid syndrome	[31]

c.345del (p.Gly116ValfsTer12)	Frameshift	∼16 years	XHIM and renal AA amyloidosis with multiples recurrent and opportunistic infections	Colombia	↓IgG/↓IgA/↑IgM	Sinopulmonary infections Nephrotic syndrome Renal AA amyloidosis Advanced kidney disease	Present case report.

## Data Availability

The data that support the findings of this study are available from the corresponding author upon reasonable request.
